# *Chryseobacterium meningosepticum* bacteremia in diabetic nephropathy patient on hemodialysis

**DOI:** 10.4103/0971-4065.73460

**Published:** 2010-10

**Authors:** M. Dias, K. Prashant, R. Pai, B. Scaria

**Affiliations:** Department of Microbiology and Nephrology, Father Muller Medical College, Mangalore, India

**Keywords:** *Chryseobacterium meningosepticum*, bacteremia, hemodialysis

## Abstract

The Chryseobacterium species are inhabitants of soil and water. In the hospital environment, they exist in water systems and wet surfaces. We report here a case of *Chryseobacterium meningosepticum* bacteremia in a diabetic nephropathy patient on hemodialysis. He was successfully treated with Vancomycin and ceftazidime for three weeks with good clinical outcome. This is the first case reported in dialysis patients from India.

## Introduction

*Chryseobacterium meningosepticum*, formerly known as Flavobacterium meningosepticum, has been reported to cause outbreaks of meningitis, primarily in premature newborns and infants in neonatal intensive care units (ICU).[1,2] In adults it can cause endocarditis, pneumonia and bacteremia, skin and soft infection.[1,3–5] There are a few reported cases of *Chryseobacterium meningosepticum* causing infection in dialysis patients.[6–9] We report here a 37-year-old with diabetic nephropathy on hemodialysis who developed bacteremia with this bacterium. Literature search showed this is the first reported case in dialysis patient from India.

## Case Report

A 37-year-old man with stage V diabetic nephropathy was admitted in the nephrology unit of a tertiary care hospital with complaints of decreased urine output, low grade fever and puffiness of face and pedal edema for one week. He is a known diabetic and hypertensive on regular treatment. He is an A.C technician by occupation, working in the Middle East. He had undergone dialysis five times in the Middle East for the same complaints. At the time of admission, he had a temperature of 100.8°F, BP – 130/90 mm Hg, Pulse -80 beats/min, Respiratory rate – 20 breaths / min. On physical examination, he had pitting pedal edema. Hemogram showed hemoglobin 8.9gm%, total count 8300/cu mm with 71 % neutrophils, 22% lymphocytes, 6% eosinophils. Other investigations showed blood urea 125 mg/dl, s. creatinine 9.4 mg/dl, S. uric acid 5.3 mg/dl, total proteins 5.0 g/dl, Albumin 2.3 g/dl, A/G ratio 0.9, Random blood sugar 110 mg/dl. HIV, HBsAg and HCV ELISA were negative.

## Microbiological Workup

The blood culture collected after dialysis grew Gram negative bacilli after 48 hours of incubation at 37°C. On blood agar, the colonies were small, convex, non hemolytic, pale yellow pigmented colonies. The Gram negative rod was non motile, catalase and oxidase positive, non nitrate reducing, OF glucose utilized oxidatively, Bile Esculin and indole positive, DNA’se negative, Arginine was dehydrolized. It did not grow at 42°C and was resistant to Polymyxin B. Based on these biochemical reactions it was identified as *Chryseobacterium meningosepticum*. Antibiotic susceptibility was done on Muller Hinton agar by Kirby-Bauer disc diffusion method. The strain was sensitive to ceftazidime, ceftriaxone, cotrimoxazole, ciprofloxacin, piperacillin–tazobactum, cefoperazone–sulbactum and vancomycin. It was resistant to ampicillin, amoxyclav, aminoglycosides, imipenem, meropenem. A repeat blood culture taken after seven days also grew *Chryseobacterium meningosepticum*. The patient was treated with vancomycin 1 gm I.V stat single dose followed by vancomycin 500 mg once every five days for four weeks and ceftazidime 1 gm I.V post dialysis, on alternate days, for three weeks according to sensitivity results. The patient became afebrile and his subsequent blood cultures were sterile. Environmental screening was done to trace the source by culturing reverse osmosis water; dialysate fluid and tap water were sterile.

## Discussion

Chryseobacterium spp are organisms of low virulence and their presence in clinical specimens usually represents colonization and not infection[1] except *Chryseobacterium meningosepticum*, which is clinically significant and known to cause variety of infections. *C. meningosepticum* infection in patients on dialysis is rare. There are a few published reports,[6,8,9] mainly reported from Asian countries. From India, most of the reported cases include meningitis[3,5] and endocarditis.[4] No reports of *C. meningosepticum* bacteremia in dialysis patients from India have been reported in English literature. There is only one report of Chryseobacterium septicemia in a renal allograft recipient.[10] Predisposing factors for *Chryseobacterium meningosepticum* infection include malignancy, neutropenia, diabetes, steroid use, malnutrition or being on dialysis. Colonization of patients through contaminated medical devices, humidifiers, incubators, intravenous catheters has been documented. They are inhabitants of soil and water and have been recovered from municipal water supplies and from hospital environment,[1] which can act as a potential source of infection resulting in outbreaks. Whenever there is an isolation of Chrysobacterium, an attempt should be made to trace the source of infection and stringent steps should be implemented to prevent the transmission of infection. Our patient had diabetic nephropathy stage V and was on regular maintenance dialysis. Our attempt to trace the source of infection was not successful as the environmental screening carried out to detect the possible source yielded negative results. The patient must have contracted the infection in Middle East where he had undergone dialysis previously.

*Chryseobacterium meningosepticum* has a peculiar antibiotic profile. The bacteria is inherently resistant to most antibiotics prescribed to treat gram negative bacteria like aminoglycosides, β-lactam agents, Chloramphenicol, carbapenems (due to the production of two betalactamases, ESBL and Class B Carbapenem, Hydrolyzing metallolacomtamase), but susceptible to agents used to treat gram positive bacteria (Rifampicin, Ciprofloxacin, Vancomycin, trimethoprim–sulfamethoxazole). Hence the appropriate choice of antibiotic for the treatment is difficult. Results of the susceptibility testing vary when different methods are used; further complicating the choice of antibiotic. The disc diffusion methods are unreliable and broth microdilution is the preferred method.[1] Though Vancomycin was used earlier to treat the patients, there are reports showing failure of this drug. Drugs like Minocyclin, trimethoprim-sulphamethoxazole and Rifampicin may be the good alternatives.[1,3,7] More studies are required for the evaluation of these drugs against *C. meningosepticum*. However, our patient responded well to Vancomycin and ceftazide.

In conclusion, *Chryseobacterium meningosepticum* can be a potential nosocomial pathogen. Positive identification of the organism enables prompt treatment and increases the chances of recovery. Administration of appropriate antibiotics, strict adherence to hand washing, routine screening of hospital water samples especially in dialysis units can prevent outbreaks with this bacteria.
